# CHIP suppresses the proliferation and migration of A549 cells by mediating the ubiquitination of eIF2α and upregulation of tumor suppressor RBM5

**DOI:** 10.1016/j.jbc.2024.105673

**Published:** 2024-01-23

**Authors:** Bo Jin, Mengran Wang, Yiheng Sun, Priscilla Ann Hweek Lee, Xiangqi Zhang, Yao Lu, Bo Zhao

**Affiliations:** Engineering Research Center of Cell and Therapeutic Antibody, Ministry of Education, and School of Pharmaceutical Sciences, Shanghai Jiao Tong University, Shanghai, China

**Keywords:** CHIP, eIF2, PERK, ubiquitination, phosphorylation, protein degradation, RBM5, ER stress

## Abstract

The protein kinase RNA-like endoplasmic reticulum kinase (PERK)–eukaryotic translation initiation factor 2 subunit α (eIF2α) pathway plays an essential role in endoplasmic reticulum (ER) stress. When the PERK–eIF2α pathway is activated, PERK phosphorylates eIF2α (p-eIF2α) at Ser51 and quenches global protein synthesis. In this study, we verified eIF2α as a bona fide substrate of the E3 ubiquitin ligase carboxyl terminus of the HSC70-interaction protein (CHIP) both *in vitro* and in cells. CHIP mediated the ubiquitination and degradation of nonphosphorylated eIF2α in a chaperone-independent manner and promoted the upregulation of the cyclic AMP-dependent transcription factor under endoplasmic reticulum stress conditions. Cyclic AMP-dependent transcription factor induced the transcriptional enhancement of the tumor suppressor genes *PTEN* and *RBM5*. Although transcription was enhanced, the PTEN protein was subsequently degraded by CHIP, but the expression of the RBM5 protein was upregulated, thereby suppressing the proliferation and migration of A549 cells. Overall, our study established a new mechanism that deepened the understanding of the PERK–eIF2α pathway through the ubiquitination and degradation of eIF2α. The crosstalk between the phosphorylation and ubiquitination of eIF2α shed light on a new perspective for tumor progression.

The endoplasmic reticulum (ER) is the largest organelle in eukaryotic cells, which plays an essential role in protein synthesis, folding, and maturation (1, 2). When unfolded or misfolded proteins accumulate in the ER, cells experience ER stress, causing them to initiate an unfolded protein response (UPR) to alleviate such stress. The UPR pathway is triggered by three ER transmembrane proteins (also known as stress sensors): inositol-requiring enzyme 1, activating transcription factor 6, and protein kinase RNA-like endoplasmic reticulum kinase (PERK) (3). Generally, these three ER stress sensors maintain their inactive state by binding to GRP78, a chaperone protein resident in the ER membrane. Once ER stress occurs, GRP78 binds to unfolded proteins and releases and activates these stress sensors (3).

The eukaryotic translation initiation factor 2 subunit α (eIF2α) is a subunit of eIF2 that is required during cap-dependent translation (4). eIF2 can form a ternary complex with GTP and an initiator tRNA (eIF2-GTP-Met-tRNAi) and then bind to a 40 s ribosomal subunit to form the 43 s preinitiation complex, which plays a crucial role in the early steps of protein synthesis (5). When the PERK–eIF2α pathway is activated, PERK phosphorylates eIF2α (p-eIF2α) at Ser51 and inhibits the exchange of eIF2α-GDP to eIF2α-GPT, which blocks preinitiation complex formation. This causes an attenuation of global protein synthesis while initiating the expression of specific genes, such as the transcriptional activator cyclic AMP-dependent transcription factor (ATF4). ATF4 induces the transcription of several genes that can rescue the cells from ER stress and also induces the phosphatase subunit GADD34 that dephosphorylates p-eIF2α as a negative feedback regulator (6).

ER stress plays an essential role in the oncogenesis and development of tumors, which enable tumors to cope with harsh living environments (7). However, prolonged and unresolved ER stress can cause tumor cell death (8). Several studies focusing on ER stress for tumor therapy have been reported. For example, bortezomib causes excessive ER stress to induce tumor cell death (9). The PERK inhibitor GSK2606414 downregulates eIF2α phosphorylation and induces tumor cell death (10). Recent studies have reported that some proteins can interact with eIF2α; for example, TIPRL can interact with eIF2α and upregulate its phosphorylation, while ERH can interact with eIF2α, and the knockdown of ERH will upregulate ATF4 and CHOP (11, 12).

Ubiquitin is a protein composed of 76 amino acids, which can be transferred to the substrate through ubiquitin-activating enzyme (E1), ubiquitin-binding enzyme (E2), and ubiquitin ligase (E3) (13). As a protein posttranslational modification process, ubiquitination is involved in many biological activities, such as cell cycle processes, endocytosis, apoptosis, signal transduction, transcriptional regulation, DNA repair, protein sorting, and protein degradation (14). However, it is difficult to identify the substrates of a specific E3 due to the crosstalk between different E3s and substrates. In our previous study, we developed an orthogonal ubiquitin transfer platform (15, 16, 17) and identified the substrates of certain E3s, including E6AP (18), carboxyl terminus of the HSC70-interaction protein (CHIP) (19), E4B (19), and Rsp5 (20). The CHIP is a U-box E3 ubiquitin ligase that interacts with chaperones by its tetratricopeptide-repeat domain and with E2-ubiquitin conjugates by its U-box domain (21). However, not all the substrates of CHIP need chaperones to facilitate their ubiquitination (22). CHIP has disparate functions in different tumors. In breast, lung, and gastric cancer, the levels of CHIP decrease and CHIP plays a role in suppressing tumorigenesis (23, 24, 25, 26, 27). Conversely, in prostate cancer, CHIP promotes tumor development (28). Some well-known proteins have been identified as substrates of CHIP, such as c-Myc, p-53, HIF-1α, and PTEN (29, 30, 31, 32). In our previous study, we employed the orthogonal ubiquitin transfer method and identified over 100 potential substrates of CHIP in HEK293 cells, including eIF2α. In this study, we verified eIF2α as a bona fide substrate of CHIP both *in vitro* and in cells. CHIP mediated the ubiquitination and degradation of eIF2α but not p-eIF2α in a chaperone-independent manner. Under ER stress, the expression levels of CHIP decreased significantly in A549 cells, following the upregulation of eIF2α, while the levels of CHIP and eIF2α remained stable in H1299 cells. CHIP overexpression in tunicamycin (TM)-stimulated A549 cells decreased eIF2α levels and induced ATF4 upregulation, which subsequently enhanced the transcription of the tumor suppressor genes such as *PTEN* and *RBM5*. Overall, this study provides new insight into the PERK–eIF2α pathway based on eIF2α ubiquitination and degradation and suggests a new role of CHIP in UPR.

## Results

### CHIP mediated the ubiquitination and degradation of eIF2α without the involvement of chaperones

**Figure 1:**
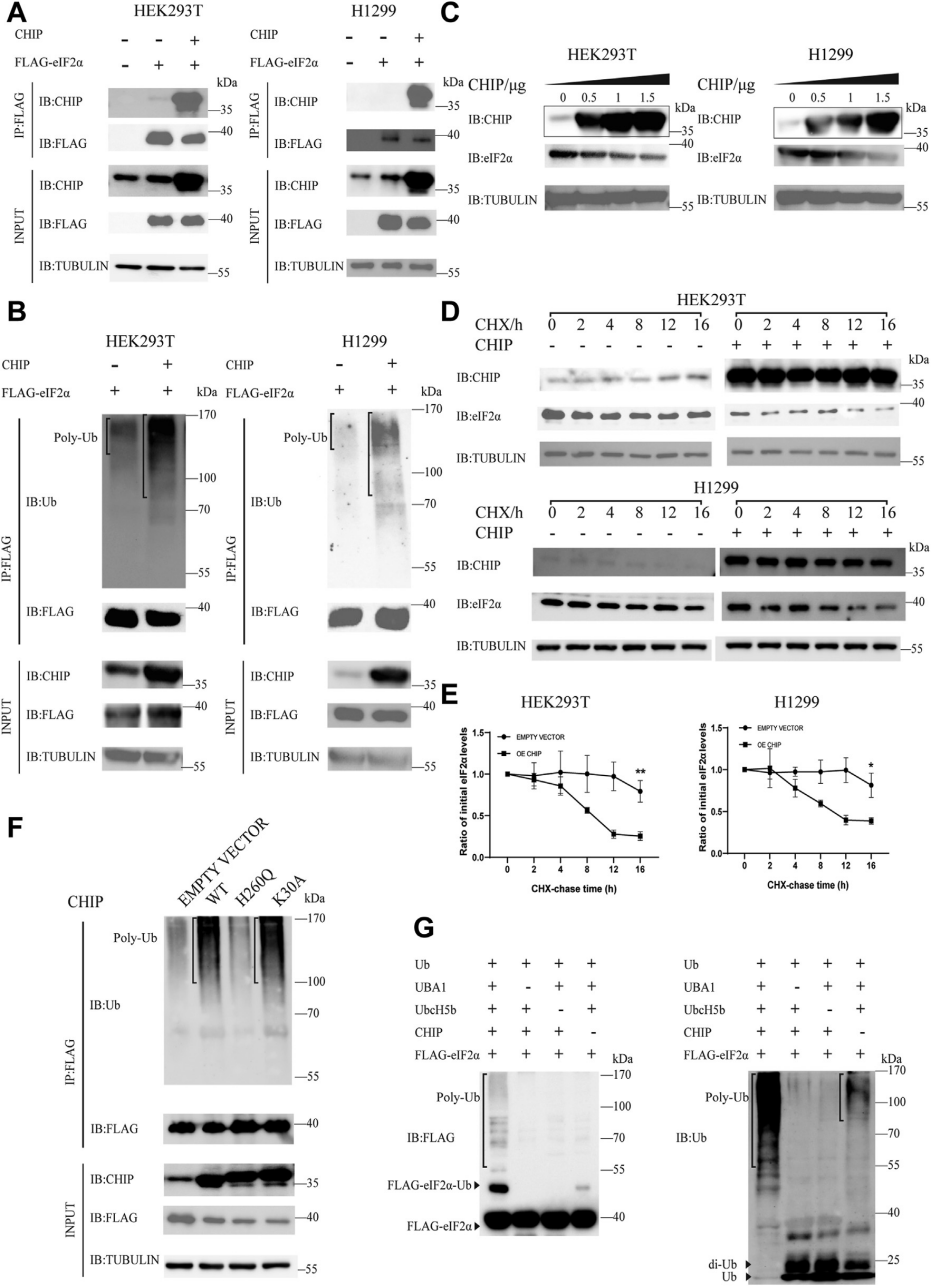
**CHIP mediates eIF2α ubiquitination and degradation in a chaperone-independent manner in HEK293T and H1299 cells.** *A*, CHIP interacts with eIF2α. HEK293T and H1299 cells are cotransfected with FLAG-*eIF2α* and exogenous *CHIP* or an empty vector as a control for 48 h, and cells without FLAG-*eIF2α* are used as a negative control. The interaction between CHIP and eIF2α is detected by a coimmunoprecipitation assay with an anti-FLAG antibody and an anti-CHIP antibody, respectively. The tubulin levels are used as an internal reference. *B*, CHIP promotes eIF2α ubiquitination. HEK293T and H1299 cells are cotransfected with FLAG-*eIF2α* and exogenous *CHIP* or an empty vector as a control for 44 h and then treated with MG132 (10 μM) for 4 h. eIF2α ubiquitination is detected by a coimmunoprecipitation assay with the anti-FLAG and anti-ubiquitin antibodies. *C*, CHIP induces eIF2α degradation. HEK293T and H1299 cells are transfected with different amounts of exogenous *CHIP* for 48 h. eIF2α degradation caused by CHIP is detected by Western blotting with the anti-CHIP and anti-eIF2α antibodies. *D*, CHIP promotes eIF2α degradation after CHX treatment. HEK293T and H1299 cells are transfected with exogenous *CHIP* or an empty vector as a control for 36 h and then treated with cycloheximide (CHX) (100 ug/ml) for indicated time points. eIF2α level changes caused by CHIP are detected by Western blotting with the anti-CHIP and anti-eIF2α antibodies. *E*, data in Figure 1*D* are quantified to show the half-lives of eIF2α in CHIP-overexpressed HEK293T and H1299 cells. A two-tailed Student’s *t* test is used and the error bars represent the SD of the mean. All statistical results are generated using GraphPad 8. Data are shown as the mean ± SD. n = 3 independent replicates. *F*, ubiquitination of eIF2α promoted by CHIP does not need a chaperone. HEK293T cells are cotransfected with FLAG-*eIF2α* and exogenous *CHIP, H260Q-CHIP, K30A-CHIP*, or an empty vector as a control for 44 h and then treated with MG132 (10 μM) for 4 h. The eIF2α ubiquitination is detected with a coimmunoprecipitation assay by using an anti-FLAG antibody and an anti-ubiquitin antibody, respectively. *G*, CHIP ubiquitinates eIF2α *in vitro*. Purified recombinant FLAG-eIF2α are incubated with or without Ube1, Ubch5b, CHIP, and ubiquitin proteins for 4 h at 37 °C. All the proteins are expressed in *Escherichia coli* cells and purified by a His tag. The ubiquitination of eIF2α is detected with an anti-FLAG antibody and an anti-ubiquitin antibody, respectively. All blots in this study are performed three independent assays with replicates showing similar results and one representative was shown in the figure. ∗*p* < 0.05; ∗∗*p* < 0.01; ∗∗∗*p* < 0.001; and n.s., not significant. CHIP, carboxyl terminus of the HSC70-interaction protein; eIF2α, eukaryotic translation initiation factor 2 subunit α.

In a previous study, we found that eIF2α may be a potential ubiquitination substrate of CHIP (19). To verify this, we first set up a coimmunoprecipitation assay to test whether CHIP and eIF2α had an interaction. HEK293T and H1299 cells were cotransfected with FLAG-tagged *eIF2α* and exogenous *CHIP*, control cells were either cotransfected with FLAG-*eIF2α* and an empty vector or just an empty vector. After the pulldown by an anti-FLAG antibody, an anti-CHIP antibody was used to detect the interaction. Since the cells contain endogenous CHIP, we saw weak bands in the control cells merely transfected with FLAG-*eIF2α* but no bands in the control cells without FLAG-*eIF2α*. However, cells cotransfected with exogenous *CHIP* and FLAG-*eIF2α* showed stronger bands, indicating there was an interaction between CHIP and eIF2α (Fig. 1*A*). To see whether CHIP can ubiquitinate eIF2α, we repeated the same assays in Figure 1*A* but replaced the anti-CHIP antibody with an anti-Ub antibody for the coimmunoprecipitation detection and found poly-ubiquitin bands formed on eIF2α; these bands were significantly stronger in the cells transfected with *CHIP*, suggesting that the ubiquitination of eIF2α was mediated by CHIP (Fig. 1, *B*). To see whether the ubiquitination mediated by CHIP will lead to the degradation of eIF2α, cells were transfected with different amounts of *CHIP* and protein levels of endogenous eIF2α were detected with an anti-eIF2α antibody. We found that the protein levels of eIF2α decreased in a dose-dependent manner; the more *CHIP* transfected, the less eIF2α remained. These results indicated that CHIP mediated the degradation of eIF2α (Fig. 1*C*). A cycloheximide (CHX)-chase assay can be used to detect protein stability. By using the protein synthesis inhibitor CHX, the total synthesis of proteins is quenched and the stability of the proteins that have been synthesized in the cells can be measured. In this study, we transfected HEK293T and H1299 cells with exogenous *CHIP* or an empty vector as a control for 36 h and then treated cells with CHX (100 μg/ml) at different time points. We observed that eIF2α stabilized after 16 h of treatment by CHX if exogenous *CHIP* was absent but was degraded in the presence of exogenous *CHIP* (Fig. 1*D*). The half-life of eIF2α was about 8 h in both cells transfected with exogenous *CHIP* (Fig. 1*E*). These results confirmed that CHIP could mediate eIF2α degradation.

As CHIP functions as an E3 depending on the presence or absence of chaperones, we wanted to know whether eIF2α ubiquitination by CHIP requires chaperone involvement. We constructed the H260Q *CHIP* mutant which disrupts the interaction between CHIP and E2s and the K30A *CHIP* mutant which disrupts the interaction between CHIP and chaperones (33, 34). Cells were cotransfected with FLAG-*eIF2α* and exogenous *CHIP, H260Q, K30A*, or an empty vector as a control for 44 h and then treated with MG132 (10 μM) for 4 h. eIF2α ubiquitination was detected by coimmunoprecipitation with an anti-FLAG antibody for pulldown and an anti-ubiquitin antibody for immunoblotting. The ubiquitination of eIF2α became weaker in *H260Q*-transfected cells but slightly weaker in *K30A-*transfected cells than that in cells transfected with the WT *CHIP* (Fig. 1*F*). These results suggested that eIF2α ubiquitination by CHIP did not require chaperones. To further confirm the ubiquitination of eIF2α mediated by CHIP in a chaperone-independent manner, we set up an *in vitro* ubiquitination assay with purified ubiquitin, UBA1 (E1), UbcH5b (E2), CHIP, and eIF2α recombinant proteins expressed in *Escherichia coli* cells. The anti-FLAG antibody was used for immunoblotting to detect the ubiquitination of eIF2α. We observed strong poly-ubiquitin bands in the presence of all the components for ubiquitin transfer. However, no poly-ubiquitin bands formed if UBA1 or UbcH5b was absent (Fig. 1*G*, left panel). For as-of-yet unknown reasons, a band appeared near the position of FLAG-eIF2α∼Ub in the CHIP-absent group (Fig. 1*G*, left panel, lane 4). In order to confirm whether this is a monoubiquitination band formed on eIF2α without CHIP, we repeated the same assay and used an anti-ubiquitin antibody to develop this blot. We observed the smear in lane 1 and the ubiquitin and diubiquitin were almost depleted, indicating strong poly-ubiquitin bands formed on eIF2α or CHIP (Fig. 1*G*, right panel, lane 1). This time, we did not observe monoubiquitination bands in the control groups (Fig. 1*G*, right panel, lanes 2–3). In the no CHIP group, E2 (UbcH5b) formed a poly-ubiquitin chain, and part of the ubiquitin and diubiquitin remained (Fig. 1*G*, right panel, lane 4). These results indicated that CHIP directly promoted eIF2α ubiquitination without chaperones *in vitro*. Taken together, we verified that eIF2α is a *bona fide* substrate of CHIP, and CHIP mediated the ubiquitination and degradation of eIF2α without chaperone involvement.

### The phosphorylation of eIF2α at Ser51 inhibited its ubiquitination and degradation mediated by CHIP

**Figure 2:**
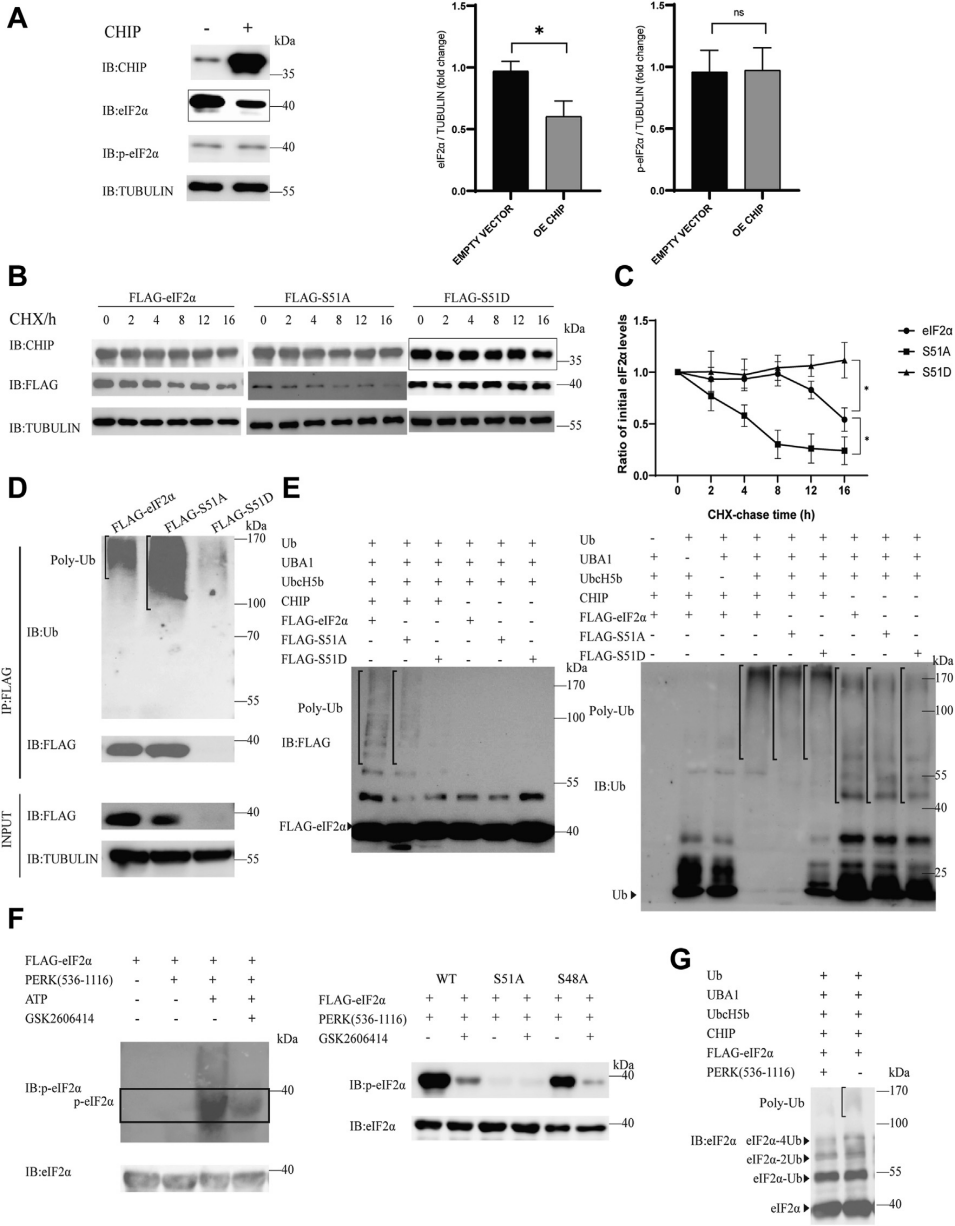
**The phosphorylation of eIF2α at Ser51 inhibits its ubiquitination and degradation mediated by CHIP.***A*, eIF2α phosphorylation inhibits its degradation. HEK293T cells were transfected with exogenous *CHIP* or an empty vector as a control for 36 h. The degradation of eIF2α and p-eIF2α caused by CHIP is detected with Western blotting by using an anti-eIF2α antibody and an anti-p-eIF2α antibody, respectively. The differences of eIF2α and p-eIF2α in two cell groups are quantified. A two-tailed Student’s *t* test was used and the error bars represent the SD of the mean. All statistical results are generated using GraphPad 8. Data are shown as the mean ± SD n = 3 independent replicates. *B*, FLAG-S51D is more stable than FLAG-eIF2α and FLAG-S51A after cycloheximide (CHX) treatment. HEK293T cells are cotransfected with exogenous *CHIP* and FLAG-*eIF2α* (WT), FLAG-*S51A*, or FLAG-*S51D* for 40 h and then treated with CHX (100 ug/ml) for indicated time points. Changes in the protein levels of FLAG-eIF2α, FLAG-S51A, or FLAG-S51D are detected with Western blotting by using an anti-FLAG antibody. *C*, data in Figure 2*B* are quantified to show the half-lives of eIF2α or mutants in CHIP-overexpressed HEK293T cells. A two-tailed Student’s *t* test is used and the error bars represent the SD of the mean. All statistical results are generated using GraphPad 8. Data are shown as the mean ± SD. n = 3 independent replicates. *D*, the ubiquitination of FLAG-S51A is stronger than that of FLAG-eIF2α. HEK293T cells are cotransfected with *CHIP* and FLAG-*eIF2α*, FLAG-*S51A*, or FLAG-*S51D* for 40 h and then treated with MG132 (10 μM) for 4 h. The ubiquitination of eIF2α, S51A, or S51D mediated by CHIP is detected by anti-FLAG and anti-ubiquitin antibodies. *E*, the ubiquitination of eIF2α, S51A, and S51D mediated by CHIP *in vitro*. Recombinant FLAG-eIF2α, FLAG-S51A, or FLAG-S51D are incubated with ubiquitin, Ube1, Ubch5b, and CHIP proteins for 4 h at 37 °C. All the proteins are expressed in *Escherichia coli* cells and purified with a His tag. The ubiquitination of eIF2α (or S51A, S51D) is detected with anti-FLAG or anti-ubiquitin antibodies. *F*, the recombinant PERK (536–1116) protein can phosphorylate eIF2α at Ser51 *in vitro*. FLAG-eIF2α, FLAG-eIF2α-S51A, and FLAG-eIF2α-S48A are incubated with ATP and PERK inhibitor GSK2607414 and with or without a recombinant PERK (536–1116) protein for 4 h at 37 °C. All the proteins are expressed in *E. coli* cells and purified with a His tag. eIF2α phosphorylation is detected by Western blotting with an anti-eIF2α antibody and an anti-p-eIF2α antibody. *G*, eIF2α phosphorylation inhibits its ubiquitination mediated by CHIP. FLAG-eIF2α is incubated with or without recombinant PERK (536–1116), and Ubiquitin, Ube1, Ubch5b, and CHIP proteins for 4 h at 37 °C. All the proteins are expressed in *E. coli* cells and purified with a His tag. The ubiquitination of eIF2α is detected by an anti-eIF2α antibody. CHIP, carboxyl terminus of the HSC70-interaction protein; eIF2α, eukaryotic translation initiation factor 2 subunit α; PERK, protein kinase RNA-like endoplasmic reticulum kinase.

eIF2α plays an essential role in ER stress response in cells. When ER stress occurs, eIF2α is phosphorylated by PERK which subsequently quenches global protein translation and initiates the transcription of specific genes, such as *ATF4*. Therefore, the phosphorylation of eIF2α is a signal of the PERK–eIF2α pathway and blocks eIF2α function. In this study, we want to see if there is a link between the ubiquitination and phosphorylation of eIF2α. HEK293T cells were transfected with either exogenous *CHIP* or an empty vector as a control for 36 h. The protein levels of eIF2α and p-eIF2α were quantified according to their Tubulin levels. It could be found that p-eIF2α was stable regardless of whether CHIP was overexpressed or not. However, eIF2α levels decreased significantly when exogenous *CHIP* was present (Fig. 2*A*). These results indicated that CHIP mainly mediated the degradation of total eIF2α, but hardly p-eIF2α. In mammalian cells, there are four eIF2α kinases: HRI, PKR, GCN2, and PERK (35, 36). Different kinases are activated under different stress conditions to initiate eIF2α phosphorylation (36). For example, PERK generally phosphorylates eIF2α under ER stress (3). It has been reported that the phosphorylation of eIF2α primarily occurs at Ser51 (37). To further verify whether eIF2α phosphorylation at Ser51 will affect its ubiquitination, we constructed the mutant *eIF2α-S51A* which cannot be phosphorylated and mutant *eIF2α-S51D* which mimics the phosphorylation of eIF2α. HEK293T cells were transfected with FLAG-tagged WT *eIF2α* (FLAG-eIF2α), mutant *eIF2α-S51A* (FLAG-S51A), or mutant *eIF2α-S51D* (FLAG-S51D), and cotransfected with *CHIP*. The cells were treated with CHX at different time points and the protein levels of eIF2α and mutants were detected. The levels of eIF2α and two mutants were quantified according to their Tubulin levels. After 16 h of treatment, both the FLAG-eIF2α and the FLAG-S51A levels decreased significantly and the half-lives were about 16 and 6 h, respectively. However, FLAG-S51D remained stable and even increased slightly after 16 h (Fig. 2, *B* and *C*). These results indicated that eIF2α phosphorylation at Ser51 enhanced its stability, and CHIP promoted the degradation of nonphosphorylated eIF2α. To further detect the difference in ubiquitination, HEK293T cells were cotransfected with *CHIP* and *FLAG-eIF2α*, *FLAG-S51A*, or *FLAG-S51D* for 40 h and then treated with MG132 (10 μM) for 4 h to block proteasome function. FLAG-tagged eIF2α or mutants were pulled down by an anti-FLAG antibody and ubiquitination was detected by an anti-Ub antibody. Clear poly-ubiquitin bands were found on eIF2α, and S51A, and S51A exhibited stronger ubiquitination than WT eIF2α (Fig. 2*D*, lanes 1–2). For as-of-yet unknown reasons, S51D expression in the cells was faint, especially when it was exposed with eIF2α and S51A on the same nitrocellulose film, therefore, the ubiquitination bands of S51D were not shown clearly (Fig. 2*D*, lane 3). To solve this problem, recombinant FLAG-eIF2α, FLAG-S51A, and FLAG-S51D proteins were expressed in *E. coli* cells and an *in vitro* ubiquitination assay was performed. The anti-FLAG antibody was used to detect the ubiquitination and the results are shown in Figure 2*E*. We observed clear poly-ubiquitin bands on eIF2α and S51A, whereas S51D ubiquitination was significantly downregulated (left panel, lane 1–3), indicating that the phosphorylation of eIF2α at Ser51 inhibited its ubiquitination. The poly-ubiquitination of eIF2α and S51A depends on CHIP, as the bands disappeared when CHIP was absent (Fig. 2*E*, left panel, lanes 4–6). To further confirm these results, we repeated the assay with an anti-ubiquitin antibody. Clear poly-ubiquitin binds were seen on eIF2α and S51A, and the ubiquitin and diubiquitin were depleted (Fig. 2*E*, right panel, lanes 4–5), while in the S51D and no-CHIP groups, we still observed the poly-ubiquitin bands because E2 (for lanes 7–9) or E3 (for lane 6) could form self-ubiquitination without E3 or the substrate (Fig. 2*E*, right panel, lanes 6–9).

To further investigate the effect of phosphorylation on ubiquitination, an *in vitro* phosphorylation assay of eIF2α was performed. PERK (536–1116), the kinase domain of full-length PERK, was expressed in *E. coli* cells and incubated with an eIF2α protein (38). An anti-eIF2α antibody was used to detect the total level of eIF2α, while an anti-p-eIF2α antibody was used to blot the phosphorylated eIF2α. We observed that PERK (536–1116) phosphorylated eIF2α in an ATP-dependent manner. Without ATP, eIF2α could not be phosphorylated (Fig. 2*F*, left panel). However, when the PERK inhibitor GSK2606414 was added to the reaction, the phosphorylation of eIF2α was markedly downregulated (Fig. 2*F*, left panel). These results demonstrated that the *in vitro* phosphorylation of eIF2α by PERK (536–1116) was successful. Although there are other potential phosphorylation sites on eIF2α, we found no reports of phosphorylation at sites other than Ser51. There is an antibody against eIF2α that is phosphorylated at Ser48 (Ser49 if the first Met is counted) in the website of Abcam (https://www.abcam.com/products/primary-antibodies/eif2s1-phospho-s49-antibody-ab131489.html); we therefore assume that the phosphorylation of eIF2α at Ser48 may have occurred. We constructed the eIF2α mutants eIF2α-S51A and eIF2α-S48A and measured the phosphorylation of WT eIF2α and two mutants mediated by PERK (536–1116). We observed that the phosphorylation of S51A almost disappeared even in the presence of PERK (536–1116); conversely, S48A could be phosphorylated well in the presence of PERK (536–1116), indicating that eIF2α phosphorylation mediated by PERK occurred on Ser51 (Fig. 2*F*, right panel). We then set up an *in vitro* ubiquitination reaction with or without PERK (536–1116). When eIF2α was blotted, clear poly-ubiquitin bands formed on eIF2α were observed in the absence of PERK (536–1116). However, the poly-ubiquitin bands weakened in the presence of PERK (536–1116); in particular, poly-ubiquitin chains formed by over four ubiquitin molecules were significantly inhibited by the phosphorylation reaction (Fig. 2*G*). Because not all of the eIF2α molecules were phosphorylated, we still observed eIF2α ubiquitination in the presence of PERK (536–1116). Taken together, we revealed that CHIP mediated the ubiquitination and degradation of nonphosphorylated eIF2α, and the phosphorylation of eIF2α at Ser51 inhibited its ubiquitination, especially the poly-ubiquitin chains formed by over four ubiquitin molecules.

### CHIP affected the transcription of tumor suppressor genes by ATF4 upregulation in A549 cells under ER stress

**Figure 3:**
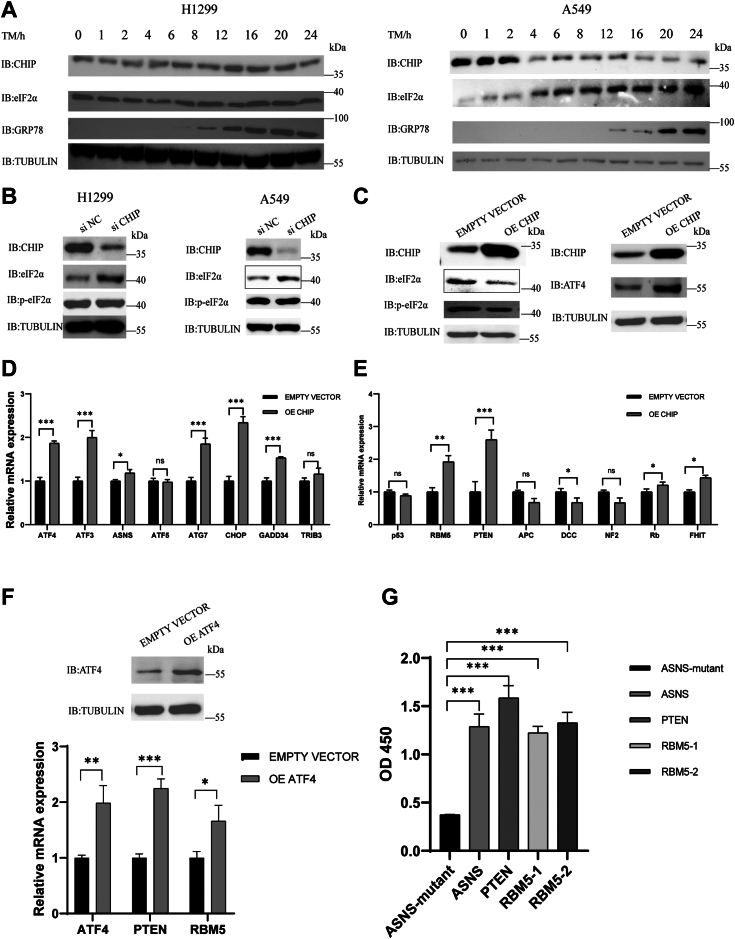
**CHIP affects the transcription of tumor suppressor genes in A549 cells under ER stress.***A*, the CHIP levels are downregulated in A549 cells under ER stress. H1299 and A549 cells are treated with TM (3 μg/ml) for indicated time points. The levels of GRP78 that act as the marker of ER stress are detected by an anti-GRP78 antibody, and the levels of CHIP and eIF2α are analyzed with an anti-CHIP antibody and an anti-eIF2α antibody, respectively. *B*, CHIP knockdown induces eIF2α upregulation. H1299 and A549 cells are transfected with si NC or si CHIP for 60 h and treated with TM (3 μg/ml) for 16 h. The expression of CHIP, eIF2α, and p-eIF2α is detected by Western blotting with anti-CHIP, anti-eIF2α, and anti-p-eIF2α antibodies, respectively. *C*, CHIP overexpression induces ATF4 upregulation and the transcription of ATF4 downstream genes. A549 cells are transfected with exogenous *CHIP* for 36 h and treated with TM (3 μg/ml) for 16 h. The expression of CHIP, eIF2α, p-eIF2α, and ATF4 is detected by Western blotting with anti-CHIP, anti-eIF2α, anti-p-eIF2α, and anti-ATF4 antibodies, respectively. *D*, Q-qPCR is used to detect the transcription of genes that are related to ER stress. *E*, Q-qPCR is used to detect the transcription of tumor suppressor genes. *F*, ATF4 induces the transcription of PTEN and RBM5. A549 cells are transfected with exogenous ATF4 for 36 h and treated with TM (3 μg/ml) for 16 h. The expression of ATF4 is detected with the anti-ATF4 antibody. Q-qPCR is used to detect the mRNA levels of *ATF4*, *PTEN*, and *RBM5*. *G*, ELISA is performed to detect the ATF4-binding elements on DNA sequences. Biotin-labeled dsDNA (200 nM) are incubated with ELISA plates, which were coated with streptavidin for 1 h at 37 °C. His-ATF4 (100 nM) is added to the plate for 1 h at 37 °C. Anti-His antibody (mouse) and anti-mouse secondary antibody (horseradish peroxidase) are used to detect the binding of ATF4 and DNA, and a TMB kit is used to measure the absorbance at 450 nm. ATF4, cyclic AMP-dependent transcription factor; CHIP, carboxyl terminus of the HSC70-interaction protein; ER, endoplasmic reticulum; eIF2α, eukaryotic translation initiation factor 2 subunit α; PERK, protein kinase RNA-like endoplasmic reticulum kinase; qPCR, real-time quantitative PCR; TM, tunicamycin.

When the PERK–eIF2α pathway is activated, eIF2α is phosphorylated by PERK and p-eIF2α inhibits the global protein synthesis but induces ATF4 upregulation. In the process of cancer cell growth, the demand for protein synthesis dramatically increases, therefore, the regulation of ER stress and the PERK–eIF2α pathway is crucial. It has been reported that CHIP functions as a tumor suppressor in lung cancer (26). To see if eIF2α ubiquitination mediated by CHIP can regulate ER stress in lung cancer cells, H1299 and A549 cells were treated with TM to induce ER stress. The GRP78 levels were measured as ER stress markers. Usually, the GRP78 levels increase after 12 h of ER stress induced by TM. We observed that the GRP78 levels increased gradually after 8 h of stimulation by TM, indicating that ER stress was induced successfully in both cancer cells (Fig. 3*A*). In H1299 cells, the levels of CHIP and eIF2α remained stable, suggesting that the CHIP–eIF2α pathway was unaffected by ER stress. Surprisingly, the CHIP levels significantly decreased over time in A549 cells; conversely, the eIF2α levels increased accordingly (Fig. 3*A*). To see whether these phenomena are cell line–specific, H1299 and A549 cells were transfected with a siRNA that can interfere with the stability of *CHIP* mRNA (siCHIP) or a scrambled siRNA (siNC) that will not bind to *CHIP* mRNA as a negative control, and the protein levels of eIF2α and p-eIF2α were detected. After 60 h of transfection, the cells were treated with TM (3 μg/ml) for 12 h. We found that compared to the siNC groups, eIF2α expression levels increased in the siCHIP groups in both cell lines, indicating that the knockdown of CHIP promoted the expression of total eIF2α (Fig. 3*B*). Conversely, in both cell lines, the protein levels of p-eIF2α remained stable in both the siNC and siCHIP groups (Fig. 3*B*). These results indicated that the CHIP–eIF2α pathway may play an essential role in both H1299 and A549 cells under ER stress conditions. However, for as-of-yet unknown reasons, CHIP expression in A549 cells was downregulated under ER stress but remained stable in H1299 cells.

When ER stress occurs, eIF2α is phosphorylated by PERK, which inhibits eIF2α functions for global protein synthesis; meanwhile, our results suggested that the remaining eIF2α would be ubiquitinated and degraded by CHIP, which could further block global protein synthesis. As CHIP is regarded as a tumor suppressor in lung cancer, we speculate that in A549 cells protein expression is abnormally enhanced, and the cells are in a state of continuous ER stress, which leads to the downregulation of CHIP expression and weakens eIF2α degradation. Therefore, eIF2α increase promotes protein synthesis and tumor growth. It has been reported that eIF2α increase can inhibit the translation of ATF4, which is a downstream signaling molecule of the PERK–eIF2α pathway (39). There are two upstream ORFs (uORFs) in the 5′ leader region of *ATF4* mRNA. uORF1 facilitates ribosome scanning and reinitiation at coding regions in the *ATF4* mRNA. When eIF2-GTP is abundant, ribosomes scan downstream of uORF1 and reinitiate at uORF2, an inhibitory element that inhibits ATF4 expression (39). In ER stress–stimulated A549 cells, CHIP expression decreased significantly. Therefore, if we increase CHIP expression levels in A549 cells, the levels of eIF2α (nonphosphorylated form) should decrease, and the amount of eIF2-GTP should also decrease, which then releases the inhibition of ATF4 expression and leads to the enhancement of the transcription of downstream genes of ATF4, such as *ATF3*, *ASNS*, *ATG7*, *CHOP*, *TRIB3*, *ATF5*, and *GADD34* (3, 40, 41, 42, 43). To test this hypothesis, A549 cells were transfected with either *CHIP* or an empty vector for 36 h and treated with TM (3 μg/ml) for 16 h. There was no difference in the expression levels of p-eIF2α in A549 cells with or without *CHIP* transfection, however, the total eIF2α levels in *CHIP*-transfected cells overexpression CHIP (OE CHIP) were lower than those in the cells transfected with an empty vector, indicating that eIF2α was degraded by exogenous *CHIP* in A549 cells (Fig. 3*C*, left panel). As the eIF2α levels affect ATF4 translation, we measured the ATF4 protein levels and found that ATF4 expression levels increased significantly in OE CHIP cells, suggesting that ATF4 upregulation was induced by eIF2α degradation mediated by CHIP (Fig. 3*C*, right panel). Next, we wanted to see whether ATF4 upregulation induces the transcription of its downstream genes. We first examined the transcription of *ATF4* by Q-PCR and found that the *ATF4* mRNA levels increased in OE CHIP cells, as expected. We then examined the transcription of ATF4 downstream genes and found that the mRNA levels of *ATF3, ASNS, ATG7, CHOP*, and *GADD34* correspondingly increased in OE CHIP cells (Fig. 3*D*). However, *ATF5* and *TRIB3* mRNA levels were unchanged in OE CHIP cells (Fig. 3*D*). Since the transcription of these two genes is also regulated by ATF4, we presume that there are other mechanisms that affect their transcription.

CHIP expression is downregulated in nonsmall cell lung cancer and CHIP works as a tumor suppressor (26). Therefore, we wanted to know whether CHIP overexpression can upregulate the transcription of tumor suppressor genes. We chose eight suppressor genes that have been reported to play an essential role in lung cancer, especially in A549 (44, 45, 46). We examined the transcription of these tumor suppressor genes by Q-PCR and found that the mRNA levels of *p53, APC, DCC*, and *NF2* slightly decreased, while those of *Rb* and *FHIT* slightly increased. However, the mRNA levels of *PTEN* and *RBM5* increased significantly in OE CHIP cells (Fig. 3*E*). Next, we wanted to confirm whether the transcriptional enhancement of *PTEN* and *RBM5* was caused by the upregulation of ATF4 expression mediated by the CHIP–eIF2α pathway. A549 cells were transfected with either an exogenous *ATF4* gene or an empty vector as a control, and the transcription of *ATF4, PTEN*, and *RBM5* was measured by Q-PCR. We observed that ATF4 expression levels increased in the cells transfected with exogenous *ATF4*, compared to those transfected with an empty vector (Fig. 3*F*). A Q-PCR assay showed that the transcription of *ATF4, PTEN*, and *RBM5* increased in OE ATF4 cells (Fig. 3*F*). These results indicated that ATF4 promoted the transcription of the tumor suppressor genes *PTEN* and *RBM5*.

Since ATF4 is a transcription factor and regulates the transcription of several genes, we wanted to determine whether there are ATF4-binding elements on *PTEN* and *RBM5* genes. We searched for gene sequences that may be ATF4-binding elements in the 2000-bp range upstream of *PTEN* and *RBM5* genes. We found a potential ATF4-binding sequence on *PTEN*: 5′- ACTGGACGTTTGTTGCAACATCGGAGAA-3′ and two potential ATF4-binding sequences on *RBM5*: 5′-CTGGTCAACATGGTGAAACCCCATCTCT-3′ and 5′-TGGAGTGCAGTGGCGCAATCTCGGCTCA-3′. It has been reported that ATF4 binds to the promoter sequence of *ASNS* at 5′-CCTCGCAGGCATGATGAAACTTCCCGCA-3′, and the mutant sequence 5′-CCTCGCAGGCATGCGCTCACTTCCCGCA-3′ will block ATF4 binding (43). Therefore, these two sequences can be used as positive and negative controls. We ordered these five ssDNA sequences with biotin labeled at 5′ termini and their reverse complementary ssDNA sequences. We performed an *in vitro* annealing assay to obtain dsDNA. Biotin-labeled dsDNA sequences were immobilized to ELISA plates, which were coated with streptavidin, and a His-tagged ATF4 protein was added to the plates. After washing, an anti-His antibody (mouse) and an anti-mouse secondary antibody horseradish peroxidase (HRP) were used to detect the binding of DNA and ATF4. We observed that ATF4 exhibited weak binding with the negative control DNA (ASNS mutant) but strong binding with the positive control DNA (ASNS) (Fig. 3*G*). ATF4 could bind to the DNA sequences of *PTEN* (PTEN) and both DNA sequences of *RBM5* (RBM5-1, RBM5-2). These results indicated that there are ATF4-binding elements on the genes of *PTEN* and *RBM5*. Taken together, we found that CHIP expression levels in A549 cells decreased significantly under ER stress conditions. Overexpression of CHIP in A549 cells reduced the levels of eIF2α by mediating its degradation, which resulted in ATF4 upregulation, and thereby induced the transcriptional enhancement of the tumor suppressor genes *PTEN* and *RBM5*.

### CHIP degraded PTEN but not RBM5 proteins in HEK 293T and A549 cells

**Figure 4:**
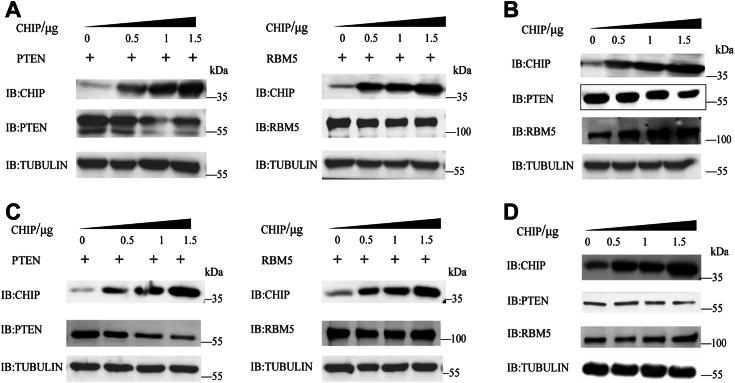
**CHIP degrades PTEN but not RBM5 proteins in HEK293T and A549 cells.***A*, CHIP degrades the exogenous PTEN protein but not the exogenous RBM5 protein in HEK293T cells. HEK293T cells are cotransfected with different amounts of CHIP with exogenous PTEN or RBM5 for 48 h. The protein levels of PTEN and RBM5 in the cells are detected with anti-CHIP, anti-PTEN, or anti-RBM5 antibodies, respectively. *B*, CHIP degrades endogenous PTEN but promotes the expression of the endogenous RBM5 protein in HEK293T cells. HEK293T cells are transfected with different amounts of *CHIP* for 48 h. Endogenous PTEN or RBM5 protein levels are detected by anti-PTEN or anti-RBM5 antibodies. CHIP levels are detected with an anti-CHIP antibody. *C*, CHIP degrades the exogenous PTEN protein but not the exogenous RBM5 protein in A549 cells. The same assays are performed as in Figure 4*A*. *D*, CHIP degrades the endogenous PTEN but promotes the expression of the endogenous RBM5 protein in A549 cells. The same assays are performed as in Figure 4*B*. CHIP, carboxyl terminus of the HSC70-interaction protein.

In Figure 3, we found that *CHIP*-overexpressed A549 cells enhanced the transcription of the tumor suppressor genes *PTEN* and *RBM5 via* the PERK–eIF2α–ATF4 pathway during ER stress. It has been reported that CHIP can ubiquitinate and degrade PTEN in HEK293T cells (32). Therefore, we wanted to see if the protein levels of PTEN and RBM5 would be affected by CHIP in the cells. We first tested the degradation of PTEN and RBM5 in HEK293T cells. HEK293T cells were cotransfected with different amounts of *CHIP* and exogenous *PTEN* or *RBM5* for 48 h. The protein levels of PTEN decreased with the increase of CHIP levels and yet RBM5 remained stable (Fig. 4*A*), demonstrating that CHIP could degrade PTEN but not RBM5. As PTEN and RBM5 were expressed by exogenous genes in this assay, we next tested the endogenous levels of PTEN or RBM5 in HEK293T cells transfected with different amounts of *CHIP*. As shown in Figure 4*B*, the protein levels of PTEN decreased with the increase of CHIP expression levels; conversely, RBM5 levels increased, consistent with the results in Figure 3*A*. We then repeated the same assays in A549 cells and achieved similar results. Exogenous PTEN degraded significantly but RBM5 remained stable in A549 cells transfected with *CHIP* (Fig. 4*C*). Although not as apparent as exogenous proteins, we still observed that endogenous PTEN was moderately degraded while endogenous RBM5 increased slightly in A549 cells (Fig. 4*D*), likely because the A549 cells were not stimulated by TM here. Taken together, we confirmed that CHIP could degrade PTEN but not RBM5 in both HEK293T and A549 cells. Although the transcription levels of *PTEN* increased in *CHIP*-transfected A549 cells after TM stimulation, the PTEN protein degraded subsequently.

### CHIP suppressed the proliferation and migration of A549 cells *via* the degradation of eIF2α and upregulation of RBM5

**Figure 5:**
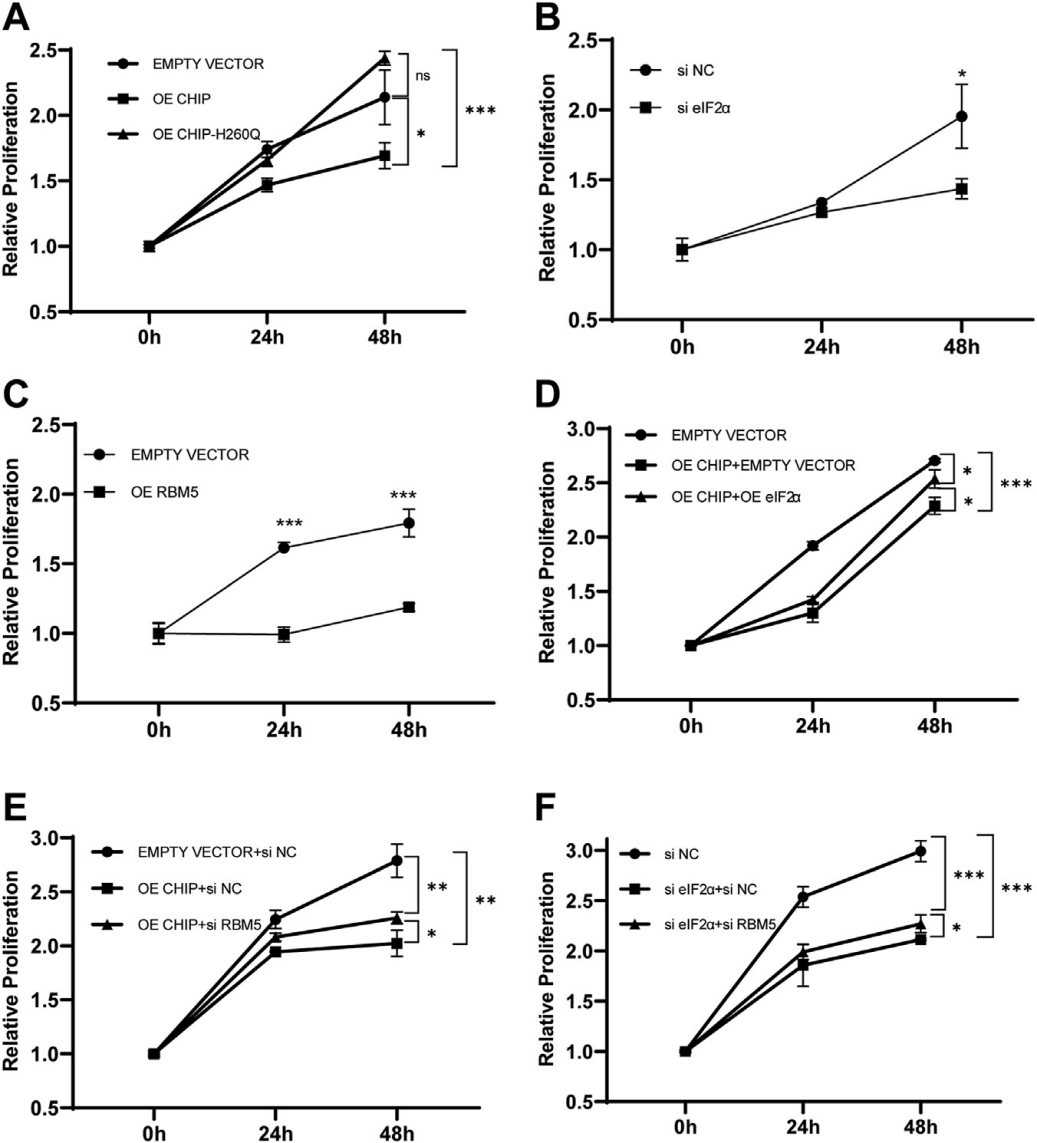
**CHIP suppresses tumor proliferation by eIF2α degradation and RBM5 upregulation.***A*, CHIP suppresses A549 proliferation. A549 cell growth curves are detected under the overexpression of CHIP or CHIP-H260Q. After 24 h, the cells are treated with TM (1 μg/ml), and the cell numbers are counted at 24 and 48 h. A CCK-8 kit was used to measure the absorbance at 450 nm. *B*, eIF2α promotes A549 proliferation. A549 cells are transfected with si NC or si eIF2α, respectively. The following assays are the same as in *A*. *C*, RBM5 suppresses A549 proliferation. A549 cells are transfected with an empty vector or *RBM5*. The following assays are the same as in *A*, *D*–*F*. CHIP suppresses the proliferation of A549 cells through the eIF2α–RBM5 pathway. Rescue assays are performed through the overexpression of eIF2α or the knockdown of RBM5 in the CHIP-overexpressed cells, or the knockdown of RBM5 in the eIF2α knockdown cells. The following assays are the same as in *A*. CHIP, carboxyl terminus of the HSC70-interaction protein; eIF2α, eukaryotic translation initiation factor 2 subunit α; TM, tunicamycin.

CHIP plays an essential role in tumor suppression in nonsmall cell lung cancer (26). We wanted to see if the CHIP-mediated degradation of eIF2α and upregulation of RBM5 contribute to tumor suppression. We first tested the proliferation of A549 cells under different conditions. A549 cells were transfected with an empty vector, exogenous *CHIP*, or the *CHIP* mutant *H260Q*. After 24 h of transfection, the cells were treated with TM (1 μg/ml), and the cell numbers were counted at 0, 24, and 48 h. Compared to the control cells (empty vector), CHIP overexpression significantly reduced A549 cell proliferation, while H260Q overexpression moderately upregulated A549 cell proliferation (Fig. 5*A*). These results indicated that the suppression of A549 cell proliferation depended on the E3 ligase activity of CHIP. Since we have confirmed that CHIP overexpression caused the degradation of eIF2α, we therefore transfected A549 cells with a siRNA that could interfere with the stability of *eIF2α* mRNA (si eIF2α) to achieve the same effect. A siRNA (siNC) that would not bind to eIF2α mRNA was used as a negative control. We found that the decrease of *eIF2α* mRNA levels due to si eIF2α could inhibit A549 cell proliferation (Fig. 5*B*). Similarly when RBM5 was overexpressed, A549 cell proliferation was also downregulated (Fig. 5*C*). To check for the presence of mechanistic links of CHIP, eIF2α, and RBM5 on the proliferation of A549 cells, we performed series rescue assays. A549 cells were transfected with an empty vector as a control, cotransfected with an empty vector and exogenous *CHIP* to overexpress CHIP (OE CHIP + empty vector), and cotransfected with exogenous *CHIP* and *eIF2α* to overexpress CHIP and eIF2α simultaneously (OE CHIP + OE eIF2α). The results are shown in Figure 5*D*. Compared to the control cells, CHIP overexpression could downregulate proliferation, which is the same as in the results in Figure 3*A*. When the CHIP-overexpressed cells were transfected with exogenous *eIF2α*, proliferation was restored (Fig. 5*D*). These results indicated that CHIP suppressed A549 cell proliferation by mediating eIF2α degradation. We continued to cotransfect A549 cells with an empty vector and a control si NC (a scrambled siRNA that does not target any mRNA of *CHIP*, *eIF2α*, or *RBM5*), then compared them to the cells cotransfected with exogenous *CHIP* and si NC (OE CHIP + si NC) and the cells cotransfected with exogenous *CHIP* and si RBM5 (OE CHIP + si RBM5). We found that si RBM5 restored the proliferation of CHIP-overexpressed cells, indicating that CHIP suppressed A549 cell proliferation by downregulating RBM5 (Fig. 5*E*). To further confirm whether CHIP downregulated RBM5 *via* the eIF2α–ATF4 pathway, we performed knockdown assays in control cells (si NC), eIF2α knockdown cells (si eIF2α + si NC), and eIF2α/RBM5 double knockdown cells (si eIF2α + si RBM5). As in the results shown in Figure 5*F*, eIF2α knockdown suppressed A549 cell proliferation, while RBM5 knockdown could restore the proliferation to a certain extent, indicating that eIF2α played a key role in A549 proliferation. Taken together, these results indicated that when CHIP levels increased in A549 cells, eIF2α degradation was promoted, and the expression of tumor suppressor RBM5 was upregulated and thus suppressed tumor proliferation.

**Figure 6:**
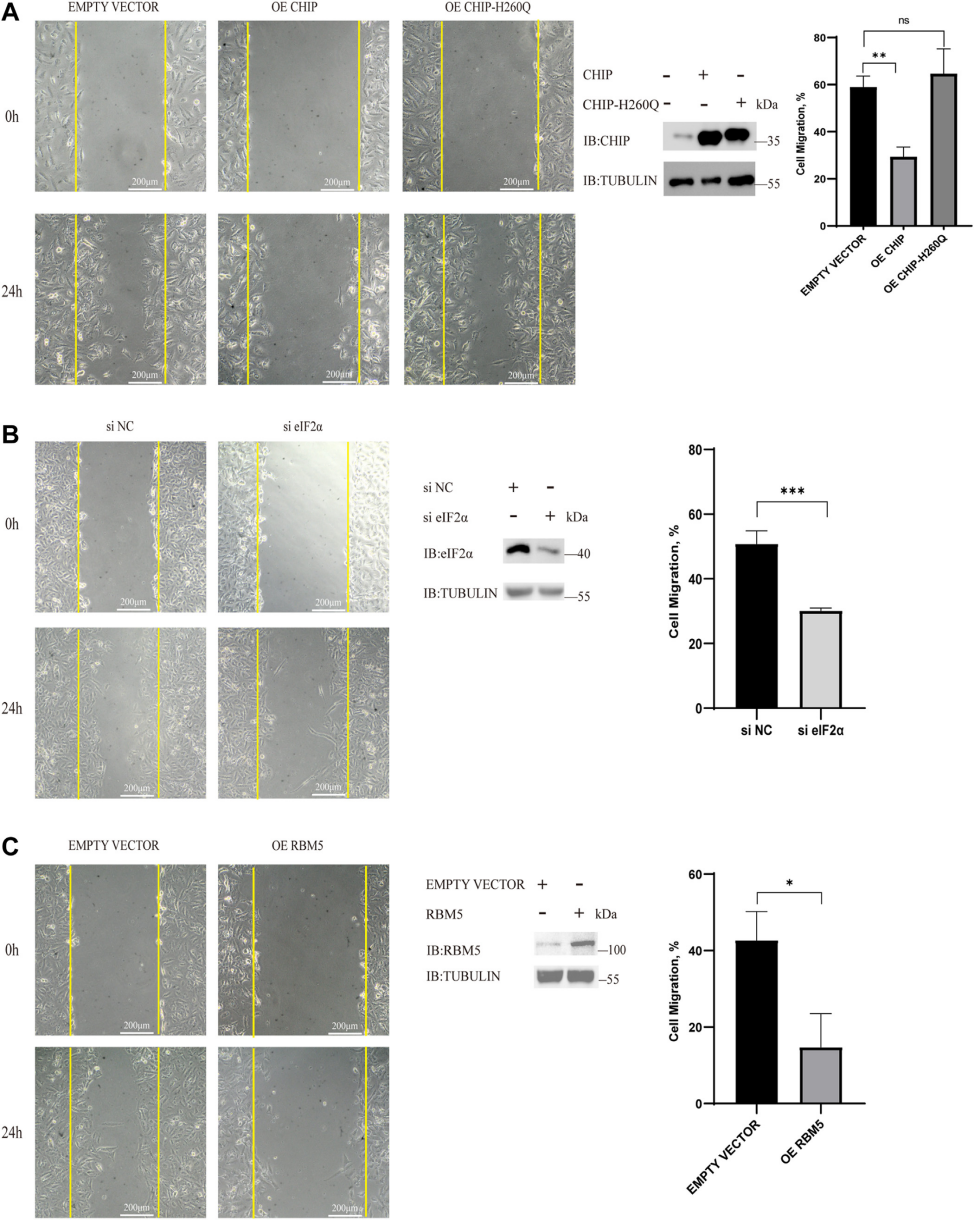
**CHIP and RBM5 suppress tumor migration, while eIF2α promotes tumor migration.***A*, CHIP overexpression can suppress the migration of A549 cells. A549 cells are transfected with exogenous *CHIP* or *CHIP-H260Q* and an empty vector as a control for 48 h. Then, the cells are treated with TM (1 μg/ml). The migration of A549 cells is detected with a scratch-wound assay. Cells are photographed (magnification: 100×) at 0 and 24 h after scratch. Histograms show the quantified data on the migration of A549 cells. *B*, interference of eIF2α expression suppresses A549 cell migration. A549 cells are transfected with si NC (a control siRNA) or si eIF2α (a siRNA for eIF2α) for 72 h. The following assays are the same as in *A*. Histograms show the quantified data on the migration of A549 cells. *C*, RBM5 overexpression suppresses A549 cell migration. A549 cells are transfected with exogenous RBM5 or an empty vector as a control for 48 h. The following assays are the same as in *A* and *B*. Histograms show the quantified data on the migration of A549 cells. CHIP, carboxyl terminus of the HSC70-interaction protein; eIF2α, eukaryotic translation initiation factor 2 subunit α; TM, tunicamycin.

**Figure 7:**
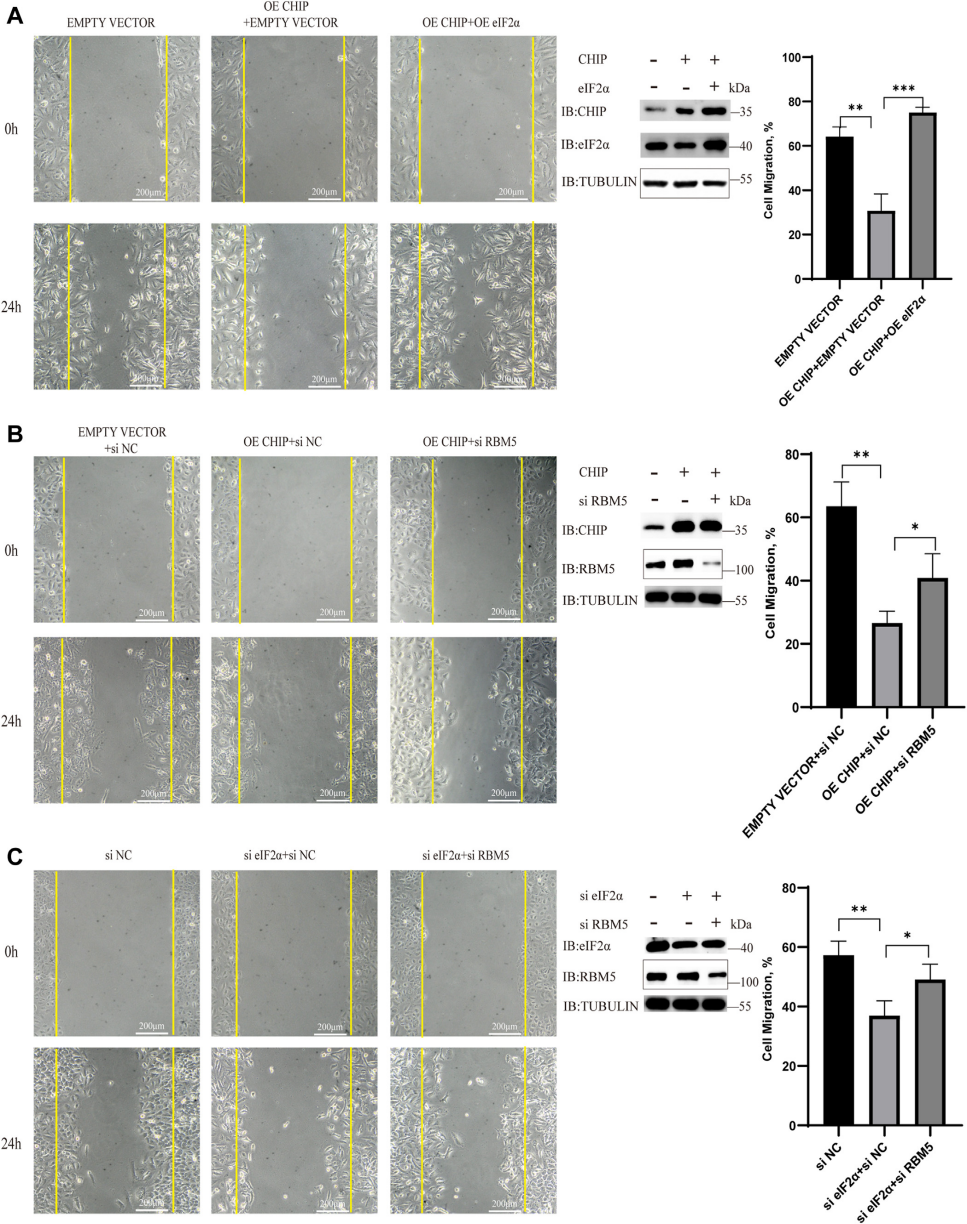
**CHIP suppresses tumor migration through eIF2α degradation and RBM5 upregulation.***A*, CHIP suppresses the migration of A549 cells while eIF2α restores it. A549 cells are transfected with exogenous CHIP or an empty vector as a control for 48 h. Then the cells are treated with TM (1 μg/ml). The migration of A549 cells is detected with a scratch-wound assay as used in Figure 6. The exogenous eIF2α is transfected into CHIP-overexpressed cells to rescue the expression of eIF2α. Histograms show the quantified data on the migration of A549 cells. *B*, RBM5 knockdown restores the migration of A549 cells. CHIP-overexpressed cells are transfected with siNC or siRBM5; the migration of A549 cells is detected. Histograms show the quantified data on the migration of A549 cells. *C*, CHIP suppresses the migration of A549 cells through the eIF2α–RBM5 pathway. A549 cells are cotransfected with si eIF2α and si RBM5 to knock down the expression of eIF2α and RBM5. Histograms show the quantified data on the migration of A549 cells. CHIP, carboxyl terminus of the HSC70-interaction protein; eIF2α, eukaryotic translation initiation factor 2 subunit α; TM, tunicamycin.

We next investigated the migration of A549 cells. A549 cells were transfected with exogenous *CHIP* or *H260Q* or an empty vector (control) for 48 h; then, the cells were treated with TM (1 μg/ml). A scratch-wound assay was used to measure migration. After 24 h, we observed a downregulation in the migration in the OE CHIP group but no significant difference was observed in the empty vector and OE H260Q groups (Fig. 6*A*). These results indicated that CHIP suppressed A549 cell migration through its E3 ligase activity. We next detected the contribution of eIF2α on migration by using RNA interference. We observed that the migration was downregulated in the si eIF2α group, indicating that the interference of *eIF2α* mRNA inhibited A549 cell migration (Fig. 6*B*). We further detected the impact of RBM5 on cell migration. A549 cells were transfected with *RBM5* and compared to the control cells, which were transfected with an empty vector. RBM5 overexpression downregulated the A549 cell migration (Fig. 6*C*). Similar to the assays on proliferation, we performed rescue assays to study the mechanistic links of CHIP, eIF2α, and RBM5 on A549 cell migration. A549 cells were transfected with an empty vector as a control, and cotransfected with an empty vector and exogenous *CHIP* to overexpress CHIP; meanwhile, the exogenous *CHIP* and *eIF2α* were cotransfected into cells to overexpress CHIP and eIF2α simultaneously. Cell migration was detected after 24 h of treatment with TM (1 μg/ml). We observed that A549 cell migration was restored when eIF2α was overexpressed, although CHIP was also overexpressed (Fig. 7*A*). These results indicated that CHIP suppressed A549 cell migration by mediating eIF2α degradation. We next cotransfected an empty vector and si NC into A549 cells as a control and compared the A549 cells to those transfected with exogenous *CHIP* and si NC (OE CHIP + si NC) and exogenous *CHIP* and si RBM5 (OE CHIP + si RBM5). The results showed that the migration of the OE CHIP group was suppressed, however, when RBM5 was knocked down by si RBM5 in OE CHIP cells, migration was restored (Fig. 7*B*). These results indicated that CHIP suppressed A549 cell migration through the downregulation of RBM5. Similarly, knockdown assays were performed, and A549 cell migration was suppressed in the si eIF2α group (si eIF2α + si NC) but restored by RBM5 knockdown (si eIF2α + si RBM5) (Fig. 7*C*). Taken together, we confirmed that CHIP suppressed A549 cell migration by mediating the degradation of eIF2α and upregulating the tumor suppressor RBM5.

## Discussion

eIF2α is a translation initiation factor that initiates 5′ cap-dependent translation (4). During ER Stress, cells downregulate global protein synthesis mainly through eIF2α phosphorylation. Currently, there are two ways to regulate eIF2α phosphorylation. One is to directly affect the function of its kinases; for example, the PERK inhibitor GSK2606414 has been used to downregulate eIF2α phosphorylation (10). The other way is to regulate the eIF2α-interacting proteins, such as TIPRL and ERH. In this study, we discovered that E3 ubiquitin ligase CHIP could ubiquitinate eIF2α during ER stress and CHIP specifically degraded nonphosphorylated eIF2α. Similar to eIF2α phosphorylation, eIF2α ubiquitination can also block eIF2α function, providing a new way to functionally regulate eIF2α.

During tumor development, the demands for protein synthesis and secretion increase, thus, tumor cells will be under continuous ER stress (7). Disruption of the adaptation of tumors to ER stress is becoming a new idea for cancer therapy. For example, excessive protein synthesis induced tumor cell death when the PERK inhibitor GSK2606414 was used to inhibit eIF2α phosphorylation (10, 47). Another example would be to increase the intensity of ER stress in cancer cells, such as by using bortezomib, a proteasome inhibitor that can potentiate ER stress by accumulating excess protein in the cells to kill tumors (9, 48). CHIP has conflicting roles in different cancer cells. For example, CHIP can inhibit tumor growth by degrading oncogenic proteins such as c-Myc, src-3, HIF-1α, and pAKT (29, 49, 50, 51). Meanwhile, CHIP also degrades some tumor suppressors such as p53 and PTEN (30, 32). CHIP expression is significantly downregulated in lung cancer, where CHIP is regarded as a tumor suppressor (26). In this study, we discovered a different antitumor mechanism of CHIP. We investigated the role of the CHIP–eIF2α pathway in two types of nonsmall cell lung cancer cell lines, H1299 and A549. After ER stress was induced, CHIP expression levels in the two cell lines significantly differed. The levels of CHIP in A549 cells decreased markedly, whereas it remained stable in H1299 cells. Although both H1299 and A549 cells are lung adenocarcinoma cells, there are many differences between them. There is no p53 protein in H1299 cells; however, A549 cells are p53-positive (52). These differences likely led to the differential responses of these two cancer cell lines to ER stress. In A549 cells, the decrease of CHIP expression levels increased eIF2α levels, thus enhancing global protein synthesis and promoting tumor growth. When the A549 cells were transfected with the exogenous *CHIP* gene to rescue CHIP expression, the levels of eIF2α decreased. The ubiquitination and degradation of eIF2α mediated by CHIP merely affected the nonphosphorylated eIF2α but not p-eIF2α, therefore, the modification would not affect the activation of the PERK–eIF2α pathway, which initiated ATF4 expression and then induced the transcription of genes to rescue the cells from ER stress. Further studies revealed that ATF4 also induced the transcription of tumor suppressor genes such as *PTEN* and *RBM5*. *PTEN* is a well-known tumor suppressor gene and has been studied extensively in recent years (53). However, a previous study has reported that PTEN is a substrate of CHIP and CHIP can promote its degradation (32). In this study, we demonstrated that *PTEN* transcription was upregulated but the PTEN protein was degraded subsequently by CHIP. RBM5 is an RNA-binding protein that can suppress tumors by arresting the cell cycle in the G1 phase and promoting cell apoptosis (44, 54). Our study showed that *RBM5* upregulation could suppress cancer cell proliferation and migration mediated by the CHIP–eIF2α pathway. This study puts forward a new mechanism of the PERK–eIF2α pathway and provides new insight into cancer therapy based on the ER stress. However, what causes the downregulation of CHIP expression in A549 cells after stimulation by TM, and why this phenomenon does not appear in H1299 cells, still merits further study.

## Experimental procedures

### Cell culture and reagents

HEK293T, H1299, and A549 cell lines were obtained from the American Type Culture Collection, and they had been verified. HEK293T cell line was cultured in Dulbecco's modified Eagle's medium (Gibco; C11995500BT) supplemented with 10% fetal bovine serum (FBS) (Hyclone; SV30087.03). A549 cell line was cultured in Dulbecco's modified Eagle's medium/F12 = 1:1 (Gibco; C11330500BT) supplemented with 10% FBS. H1299 cell line was cultured in RPMI1640 (Gibco; C11875500BT) supplemented with 10% FBS. All cells were cultured at 37 °C with 5% carbon dioxide.

Reagents: IPTG (Sangon Biotech; A100487-0005), Kanamycin (Beyotime; A506636-0025), MG132 (MCE; HY-13259), TM (Beyotime; SC0393-10 mM), ATP (Sangon Biotech; A600020), GSK2606414 (MCE; HY-18072), and CHX (MCE; HY-12320).

### siRNA and antibodies

All the siRNA were ordered from Sangon Biotech. The siRNA sequences used in the manuscript were below:

si eIF2α: 5′-GCCCAUUAAGAUUAAUCUAAUTT-3′

si CHIP: 5′-GACGCAUUCAUCUCUGAGAAUTT-3′

si RBM5: 5′-GCUGGAGGAUUGGAAUCUGAUTT-3′

The following antibodies were used for Western blotting. anti-CHIP (Abcam, ab134064, 1:5000), anti-FLAG (Sigma, F3165, 1:1000), anti-Tubulin (Abmart, M2005S, 1:2000), anti-ubiquitin (Abcam, ab223613, 1:5000), anti-eIF2α (Proteintech, 11170-1-AP, 1:1000), anti-p-eIF2α (Abcam, ab32157, 1:2500), anti-GRP78 (Abcam, ab108613, 1:5000), anti-PTEN (Abcam, ab170941, 1:5000), anti-RBM5 (Proteintech, 19930-1-AP, 1:1000), anti-ATF4 (Proteintech, 60035-1-Ig, 1:2500), and anti-His(Cell Signaling Technology, 2366S, 1:1000).

### Construction of the plasmids

PLVX-Mcherry-CHIP, PLVX-Mcherry-eIF2α-FLAG, pET28a-Ubiquitin, pET28a-Uba1, pET28a-Ubch5b, pET28a-CHIP, and pET28a-Ub were originally stored in the laboratory. The inserts of the constructs were amplified by PCR, and a complementary DNA (cDNA) library derives from HEK293T was used as template. After double digestion, the PCR product was cloned into their corresponding vectors. The PCR primers used in this study are list in Table S1.

### Cell transfection

Cells were plated within 24 h prior to transfection. HEK293T cells were transfected with Polyethylenimine Linear (PEI) MW40000 Kit (Yeasen; 40816ES03). H1299 and A549 cells were transfected with Lipofectamine 3000 (ThermoFisher; L3000075) Kit.

### Recombinant protein expression and purification

Recombinant proteins were expressed in *E. coli* cells. The pET28a + plasmid was used to clone the genes for protein expression. The recombinant pET28a + plasmids were transformed into BL21 chemical competent cells, and the cells were plated on the LB-agar plates with 50 μg/ml kanamycin. The single clone from the plates was transferred to 5 ml of 2XYT (1.6% (W/V) tryptone, 1% (W/V) yeast extract, 0.5% (W/V) NaCl) medium containing 50 μg/ml kanamycin. After incubating on a shaking incubator at 37 °C for approximately 10 h, it was added to 1 L of 2XYT medium containing 50 μg/ml kanamycin and continued to grow on a shaking incubator at 37 °C. Once the *A*_600_ reached 0.8 to 1, the *E. coli* cells were induced with 1 mM IPTG at 20 °C for 16 h. The cells were collected and centrifuged at 5000 rpm at 4 °C. Cell pellets were lysed in lysis buffer (50 mM Tris Base, 500 mM NaCl, 5 mM imidazole, pH = 8), and the cell lysate were centrifuged at 17,000 rpm at 4 °C. The supernatants were bound to Nuvia immobilized metal ion affinity chromatography resin (Bio-Rad; 780-0800) for 2 h at 4 °C in a shaker, and transferred into a gravity column. After washing by wash buffer (50 mM Tris Base, 500 mM NaCl, 20 mM imidazole, pH = 8), proteins were eluted with elution buffer (50 mM Tris Base, 500 mM NaCl, 250 mM imidazole, pH = 8) and dialyzed overnight to remove salt.

### Coimmunoprecipitation

Mammalian cells were lysed by radio immunoprecipitation assay lysis buffer I (Sangon Biotech, C500005-0100), containing Protease Inhibitor Cocktail (MCE; HY-K0010), PMSF (Beyotime, ST507-10 ml), 100X Phosphatase inhibitor complex I (Sangon Biotech; C500017-0001). 1 mg cell lysate was mixed with 10 μl anti-Flag M2 agarose gel (Merck; A2220-10 ML) and incubated in 4 °C for 4.5 h. Unbound proteins were washed with tris buffered saline (TBS) (Sangon Biotech, B040126-0005). The mixture was boiled in SDS-PAGE loading buffer (with DTT, Sangon Biotech, C508320-0010) and analyzed by Western blotting.

### Ubiquitylation assay in the cell

Mammalian cells were transfected with indicated plasmids for 44 h and treated with 10 μM of the proteasome inhibitor MG132 for 4 h. The cells were lysed in radio immunoprecipitation assay lysis buffer I. One milligram lysate was immunoprecipitated with 10 μl anti-Flag M2 agarose gel and incubated for 5 h in 4 °C. After washing with TBS, the proteins were released and boiled in SDS-PAGE loading buffer and analyzed by Western blotting with an anti-ubiquitin antibody and an anti-FLAG antibody.

### *In vitro* ubiquitylation assay

50 μM Ubiquitin, 1 μM Uba1, 10 μM Ubch5b, 5 μM CHIP, and 10 μM flag-tagged eIF2α (or mutants) were incubated with 20 mM DTT, 20 mM ATP, 20 mM MgCl_2_ in TBS buffer for 2 h at 37 °C. The mixture was boiled in SDS-PAGE loading buffer and analyzed by Western blot and immunoblotting with anti-Flag antibody and anti-Ub antibody.

### *In vitro* kinase assay

10 μM Flag-tagged eIF2α was incubated with or without 1 μM PERK (536–1116), 0.01 mM ATP, and 0.5 μM GSK2606414 in kinase reaction buffer (2 mM DTT, 5 mM MgCl2) and PBS 2 h at 37 °C. The mixture was boiled in SDS-PAGE loading buffer and analyzed by immunoblotting with anti-eIF2α antibody and anti-p-eIF2α antibody.

### Quantitative real-time PCR

A549 cells were transfected with CHIP, ATF4, or an empty vector for 36 h and treated with TM (3 μg/ml) for 16 h. Then cells were treated with TRIzol reagent (Invitrogen; 15596026), followed by total RNA isolation using the standard protocol. RNA was reverse-transcribed into cDNA using the ReverTra AceTM qPCR RT Kit (TOYOBO, FSQ101). Quantitative PCR was performed for target gene expression analysis using the TOROGreenqPCR Master Mix (TOROIVD, QST-100). Actin was measured as a reference gene. All the primers used in this study were listed in Table S2.

### Cell proliferation assay

A549 cells were seeded in a 6-well plate and allowed confluency around 70%. The cells were transfected with overexpression plasmids or siRNAs as main text described. After 24 h, cells were plated in 96-well plates (100 μl cell suspensions, 2 × 10^4^ cells/ml). Each cell group was seeded in three 96-well plates to achieve three time points of measurement. The cells were treated with TM (3 μg/ml). Cell Counting Kit-8 (Biosharp; BS350A) was used to measure the cell viability at different time points. The plates were incubated at 37 °C for 1.5 h, and the absorbance at 450 nm was measured.

### Cell migration assay

A549 cells were seeded in a 6-well plate and allowed confluence near 100%. The cells were cultured in 1% FBS medium containing TM (1 μg/ml). A sterile pipette tip was used to make the scratch. The photographs were took with a 100x microscope equipped a camera and the proportion of cell migration was calculated.

### DNA and protein-binding assay

A His-ATF4 protein was expressed in *E. coli* cells. The 5′-terminal biotin-labeled ssDNA and their complementary sequences were synthesized. The sequences were listed in Table S3. ssDNA and corresponding cDNA were mixed in equal proportion at 95 °C for 3 min, then annealed at room temperature to obtain dsDNA. The biotin-labeled dsDNA (200 nM) was bound to ELISA plates, which were coated with streptavidin for 1 h at 37 °C, and then a His-ATF4 protein (100 nM) was added to incubate 1 h at 37 °C. An anti-His antibody (mouse) was used to test the binding of ATF4 and DNA and a secondary antibody (anti-Mouse) containing HRP was added to bind the anti-His antibody. After adding HRP substrate TMB, absorption at *A*_450_ was detected.

### Statistical analysis

For Figures 1*E* and 2, *A*, and *C*, we analyzed the grayscale intensities of the eIF2α (or p-eIF2α) and Tubulin bands in the Western blot figures with ImageJ (https://imagej.net/software/imagej/). The relative grayscale intensities of each eIF2α band were calculated based on the corresponding Tubulin intensities at different time points and compared to the intensity at time zero (0 h) to achieve the relative abundance. For real-time quantitative PCR in Figure 3, *D*–*F*, we calculated the relative levels of the target gene compared to actin in each sample and calculated the differences in gene expression between the overexpression group and the control group. For Figure 3*G*, the statistical analysis based on the absorbance values at *A*_450_ for each group. For Figure 5, the transfected cells were seeded in 96-well plates (100 μl cell suspension, 2 × 10^4^ cells/ml). After cell adhesion, the culture medium was added TM (3 μg/ml). A Cell Counting Kit-8 (Biosharp; BS350A) was used to quantify the cell number at different time points. Relative proliferation = final number of cells/initial number of cells. The final cell number means the cell quantity after 24 h or 48 h, and the initial cell number means the cell quantity at 0 h when TM was added. The experiments were repeated at least three times. For Figures 6 and 7, the transfected cells were cultured in a medium containing 1% FBS and TM (1 μg/ml). When cell confluence was nearly 100%, a sterile pipette tip was used to make a scratch. After washing by PBS, the cells were photographed at different time points. The area of wound healing was quantified and compared to the baseline values, and the results were the cell migration percent. Cell migration = migration area/scratched area. The migration area means the area that cell migrates over 24 h, and the scratched area means the area that scratched at 0 h. The experiments were repeated at least three times. Two-tailed Student’s *t* test was used to show the statistical significance of two groups, and the error bars represent SD of the mean (SD). All statistical results are generated by GraphPad 8 (https://www.graphpad.com). Data are shown as mean ± SD. ∗, *p* < 0.05; ∗∗, *p* < 0.01; ∗∗∗, *p* < 0.001; and n.s., not significant.

## Data availability

The data that support the findings of this study are available on request from the corresponding author.

## Supporting information

This article contains Supporting information.

## Conflict of interest

The authors declare that they have no conflicts of interest with the contents of this article.
