# Supplementary material for: CHIP suppresses the proliferation and migration of A549 cells by mediating the ubiquitination of eIF2α and upregulation of tumor suppressor RBM5
Source: J Biol Chem. 2024 Jan 23;300(3):105673. doi: 10.1016/j.jbc.2024.105673 (PMC10877634; doi:10.1016/j.jbc.2024.105673)
Supplement: Supplemental information [file mmc1.docx]

**Table S1.** **Primer sequences used for PCR**

| Primer name | Function | Sequence |
| --- | --- | --- |
| BJ29 | PLVX-CHIP-Forward | 5’-GGAGCGCCAGAATTCATGAAGGGCAAGGAGGAG -3’ |
| BJ32 | PLVX-CHIP-Reverse | 5’-TAGGATGGATCCTCAAGCGTAGTCTGGGACGTCGTATGGGTAGTA  GTCCTCCACCCAGC-3’ |
| BJ33 | PLVX-CHIP-H260Q-Reverse | 5’-AAAATGACCCACACGCTGCAGTTGCTCCTCGATGTCCT-3’ |
| BJ34 | PLVX-CHIP-H260Q- Forward | 5’-AGGACATCGAGGAGCAACTGCAGCGTGTGGGTCATTTT-3’ |
| BJ35 | PLVX-CHIP-K30A-Reverse | 5’-AACAGACGATTGCCCTGCTCTGCGAGCTCCTGCGCGCT -3’ |
| BJ36 | PLVX-CHIP-K30A-Forward | 5’-AGCGCGCAGGAGCTCGCAGAGCAGGGCAATCGTCTGTT -3’ |
| BJ37 | pET28a-PERK(536-1116)-Forward | 5’-GAGGATGCTAGCACAACGTTTATTGTGCGCAG -3’ |
| BJ38 | pET28a-PERK(536-1116)-Reverse | 5’-GTCCTACTCGAGCTAATTGCTTGGCAAAGGGCTAT-3’ |
| BJ39 | pET28a-eIF2α-Forward | 5’-TAGGATGCTAGCATGCCGGGTCTAAGTTGTAG-3’: |
| BJ40 | pET28a-eIF2α-Reverse | 5’-AGCCTGCTCGAGTTACTTATCGTCATCGTCTTTG -3’: |
| BJ42 | PLVX-RBM5-Forward | 5’-AGCGTCGAATTCATGGGTTCAGACAAAAGAG-3’ |
| BJ44 | PLVX-RBM5-Reverse | 5’-TGAACCTCTAGACTCCATCTCAGTGAACC-3’ |
| BJ49 | PLVX-PTEN-Forward | 5’-TTCCGAGAATTCATGACAGCCATCATCAAAGA -3’ |
| BJ50 | PLVX-PTEN-Reverse | 5’-GCGCGGTCTAGAGACTTTTGTAATTTGTGTATG-3’ |
| BJ53 | PLVX-ATF4-Forward | 5’-CTCCAGGAATTCATGACCGAAATGAGCTTCCTGAG-3’ |
| BJ54 | PLVX-ATF4-Reverse | 5’-TTCAATGGATCCTCAAGCGTAGTCTGGGACGTCGTATGGGTAGG  GGACCCTTTTCTTCCCCCTT-3’ |
| BJ55 | pET28a-eIF2α-S49AForward | 5’-AGGCATGATTCTTCTTGCTGAATTATCCAGAAGGCGT-3’ |
| BJ56 | pET28a-eIF2α-S49A- Reverse | 5’-ACGCCTTCTGGATAATTCAGCAAGAAGAATCATGCCT-3’ |
| W1 | PLVX-eIF2α-Forward | 5’-CCTGCCGAATTCATGCCGGGTCTAAGTTGTA-3’ |
| W2 | PLVX-eIF2α-Reverse | 5’-CGGAGCGGATCCTTACTTATCGTCATCGTCTTTG-3’ |
| W3 | PLVX-eIF2α-S51A- Forward | 5’-GTGAATTAGCCAGAAGGCGTATCCG-3’ |
| W4 | PLVX-eIF2α-S51A- Reverse | 5’-CGGATACGCCTTCTGGCTAATTCAC-3’ |
| W5 | PLVX-eIF2α-S51D- Forward | 5’-GTGAATTAGACAGAAGGCGTATCCG-3’ |
| W6 | PLVX-eIF2α-S51D- Reverse | 5’-CGGATACGCCTTCTGTCTAATTCAC-3’ |

**Table S2. Primer sequences used for qPCR**

| Primer name | Function | Sequence |
| --- | --- | --- |
| ATF4-F | ATF4-Forward | 5’-CTCCGGGACAGATTGGATGTT-3’ |
| ATF4-R | ATF4-Reverse | 5’-GGCTGCTTATTAGTCTCCTGGAC-3’ |
| CHOP-F | CHOP-Forward | 5’-GGAAACAGAGTGGTCATTCCC-3’ |
| CHOP-R | CHOP-Reverse | 5’-CTGCTTGAGCCGTTCATTCTC-3’ |
| ATF3-F | ATF3-Forward | 5’-CCTCTGCGCTGGAATCAGTC-3’ |
| ATF3-R | ATF3-Reverse | 5’-TTCTTTCTCGTCGCCTCTTTTT-3’ |
| ATG7-F | ATG7-Forward | 5’-ATGATCCCTGTAACTTAGCCCA-3’ |
| ATG7-R | ATG7-Reverse | 5’-CACGGAAGCAAACAACTTCAAC-3’ |
| ATF5-F | ATF5-Forward | 5’-AGGGGACCGCAAGCAAAAG-3’ |
| ATF5-R | ATF5-Reverse | 5’-GCCTTGTAAACCTCGATGAGC-3’: |
| ASNS-F | ASNS-Forward | 5’-ATCACTGTCGGGATGTACCC-3’ |
| ASNS-R | ASNS-Reverse | 5’-CTTCAACAGAGTGGCAGCAA-3’ |
| β-ACTIN-F | β-ACTIN-Forward | 5’-CATGTACGTTGCTATCCAGGC-3’ |
| β-ACTIN-R | β-ACTIN-Reverse | 5’-CTCCTTAATGTCACGCACGAT-3’ |
| GADD34-F | GADD34-Forward | 5’-AGCCACGGAGGATAAAAGAACA-3’ |
| GADD34-R | GADD34-Reverse | 5’-CTGAACGATACTCCCAGGACC-3’ |
| TRIB3-F | TRIB3-Forward | 5’-AAGCGGTTGGAGTTGGATGAC-3’ |
| TRIB3-R | TRIB3-Reverse | 5’-CACGATCTGGAGCAGTAGGTG-3’ |
| RBM5-F | RBM5-Forward | 5’-ACCGATCCGAAGATGGCTAC-3’ |
| RBM5-R | RBM5-Reverse | 5’-CTCTCCCTCTCGTCACTGATG-3’ |
| PTEN-F | PTEN-Forward | 5’-TTTGAAGACCATAACCCACCAC-3’ |
| PTEN-R | PTEN-Reverse | 5’-ATTACACCAGTTCGTCCCTTTC-3’ |
| p53-F | p53-Forward | 5’-GAGGTTGGCTCTGACTGTACC-3’ |
| p53-R | p53-Reverse | 5’-TCCGTCCCAGTAGATTACCAC-3’ |
| APC-F | APC-Forward | 5’-AAAATGTCCCTCCGTTCTTATGG-3’ |
| APC-R | APC-Reverse | 5’-CTGAAGTTGAGCGTAATACCAGT-3’ |
| DCC-F | DCC-Forward | 5’-GACTTTACCAATGTGAGGCATCT-3’ |
| DCC-R | DCC-Reverse | 5’-GGTCCTGCTACTGCAACTTTT-3’ |
| NF2-F | NF2-Forward | 5’-TTGCGAGATGAAGTGGAAAGG-3’ |
| NF2-R | NF2-Reverse | 5’-CAAGAAGTGAAAGGTGACTGGTT-3’ |
| Rb-F | Rb-Forward | 5’-CTCTCGTCAGGCTTGAGTTTG-3’ |
| Rb-R | Rb-Reverse | 5’-GACATCTCATCTAGGTCAACTGC-3’ |
| FHIT-F | FHIT-Forward | 5’-ATCTCATCAAGCCCTCTGTAGT-3’ |
| FHIT-R | FHIT-Reverse | 5’-GGACGCAGGTCATGGAAGC-3’ |

**Table S3. Single-stranded DNA (forward) and complementary DNA (reverse) sequences**

| DNA name | Function | Sequence |
| --- | --- | --- |
| AsnS-a | AsnS-Forward | 5’-CCTCGCAGGCATGATGAAACTTCCCGCA-3’ |
| AsnS-b | AsnS-Reverse | 5’-TGCGGGAAGTTTCATCATGCCTGCGAGG-3’ |
| PTEN-a | PTEN-Forward | 5’-ACTGGACGTTTGTTGCAACATCGGAGAA-3’ |
| PTEN-b | PTEN-Reverse | 5’-TTCTCCGATGTTGCAACAAACGTCCAGT-3’ |
| RBM5-a1 | RBM5-1-Forward | 5’-CTGGTCAACATGGTGAAACCCCATCTCT-3’ |
| RBM5-b1 | RBM5-1-Reverse | 5’-AGAGATGGGGTTTCACCATGTTGACCAG-3’ |
| RBM5-a2 | RBM5-2- Forward | 5’-TGGAGTGCAGTGGCGCAATCTCGGCTCA-3’ |
| RBM5-b2 | RBM5-2-Reverse | 5’-TGAGCCGAGATTGCGCCACTGCACTCCA-3’ |
| AsnS-mutant-a | AsnS-mutant-Forward | 5’-CCTCGCAGGCATGCGCTCACTTCCCGCA-3’ |
| AsnS-mutant-b | AsnS-mutant-Reverse | 5’-TGCGGGAAGTGAGCGCATGCCTGCGAGG-3’: |
